# Optimizing extubation success: a comparative analysis of time series algorithms and activation functions

**DOI:** 10.3389/fncom.2024.1456771

**Published:** 2024-10-04

**Authors:** Kuo-Yang Huang, Ching-Hsiung Lin, Shu-Hua Chi, Ying-Lin Hsu, Jia-Lang Xu

**Affiliations:** ^1^Division of Chest Medicine, Department of Internal Medicine, Changhua Christian Hospital, Changhua, Taiwan; ^2^Institute of Genomics and Bioinformatics, National Chung Hsing University, Taichung, Taiwan; ^3^Ph.D. Program in Medical Biotechnology, National Chung Hsing University, Taichung, Taiwan; ^4^Respiratory Therapy Section for Adult, Changhua Christian Hospital, Changhua, Taiwan; ^5^Department of Applied Mathematics, Institute of Statistics, National Chung Hsing University, Taichung, Taiwan; ^6^Department of Computer Science and Information Engineering, Chaoyang University of Technology, Taichung, Taiwan

**Keywords:** time series, deep learning, extubation, weaning, smart healthcare

## Abstract

**Background:**

The success and failure of extubation of patients with acute respiratory failure is a very important issue for clinicians, and the failure of the ventilator often leads to possible complications, which in turn leads to a lot of doubts about the medical treatment in the minds of the people, so in order to increase the success of extubation success of the doctors to prevent the possible complications, the present study compared different time series algorithms and different activation functions for the training and prediction of extubation success or failure models.

**Methods:**

This study compared different time series algorithms and different activation functions for training and predicting the success or failure of the extubation model.

**Results:**

The results of this study using four validation methods show that the GRU model and Tanh’s model have a better predictive model for predicting the success or failure of the extubation and better predictive result of 94.44% can be obtained using Holdout cross-validation validation method.

**Conclusion:**

This study proposes a prediction method using GRU on the topic of extubation, and it can provide the doctors with the clinical application of extubation to give advice for reference.

## 1 Introduction

With the aging of the population and the continuous improvement of medical technology, the number of patients requiring mechanical ventilation is gradually increasing (Criner, 2012). In the United States, there is an analysis of ICU occupancy and utilization and it was found that there is sufficient capacity to care for the patients (Wunsch et al., 2013), and the daily cost of patients requiring mechanical ventilation is as high as US$2,278 (Cooper and Linde-Zwirble, 2004).

In Taiwan, the number of patients requiring mechanical ventilation is increasing which will also lead to an increase in healthcare costs (Cheng et al., 2008). Therefore, the National Health Insurance Board (NHIB) has established a requirement for patients in respiratory care to be transferred to a respiratory care center after 21 days in the ICU and to a respiratory care unit after 42 days in the respiratory care center. Only a minority of patients admitted to a respiratory care unit can be extubated and discharged from the hospital (Hui et al., 2010), and only half of the patients in respiratory care centers can be extubated (Cheng et al., 2007), while one-third of patients in ICUs are mechanically ventilated (Cohen et al., 2019), and 10–20% of extubated patients may need to be reintubated, which is associated with a six-fold mortality rate (Tobin, 2001).

Rapid Shallow Breathing Index (RSBI) is an important indicator of extubation, and RSBI is usually measured at the beginning of the Spontaneous Breathing Trial (SBT), and RSBI at the completion of the SBT can be an effective predictor of extubation (Kuo et al., 2006), and the Convolutional Nerve Network (CNN) can be utilized in the off-respirator with clinical significance and appropriate features to achieve good results (Jia et al., 2021).

In this study, we used every second data available on the ventilator (including: Vte, RR, Ppeak, Pmean, PEEP, FiO2) to advise the doctor when the patient is ready to be extubated, and the LSTM can be used to effectively determine the patient’s advice on the success or failure of extubation.

The remainder of this article is as follows: Section 2 reviews the literature on deep learning about RNN, LSTM and GRU and. Section 3 introduces the proposed research framework and methods. Section 4 discusses research results and performance evaluation. Section 5 is discussion. Section 6 is conclusion.

## 2 Related work

### 2.1 Deep learning

#### 2.1.1 Recurrent neural networks

**FIGURE 1:**
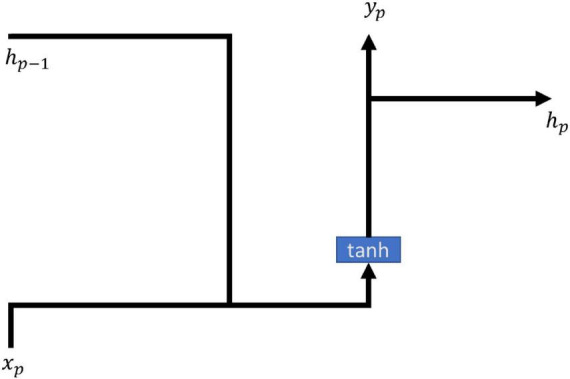
RNN architecture.

Recurrent neural networks (RNN) is a way of cycling the state of the self through its own network, and the method allows the transmitted message to survive and be input in the structure of the time series, while RNNs are elusive in terms of long term memory effects, often due to gradient disappearance leading to the long term meaning to be affected by the short term memory. RNN models are often used in many medical fields such as snoring and non-snoring prediction (Arsenali et al., 2018), hemoglobin values in end-stage renal patients (Lobo et al., 2020), and septic symptoms prediction (Scherpf et al., 2019). All of them can be predicted using RNN modeling with good results. The main formulas of the model of RNN are shown in (1, 2), and the architecture is shown in Figure 1.


(1) $$h_{p}=\text{tanh}\,\left(W_{h}\,h_{p-1}+W_{x}\,X_{t}\right)$$

(2) $$y_{p}=g\,\left(w_{y}*h_{p}\right)$$

#### 2.1.2 Long short-term memory

**FIGURE 2:**
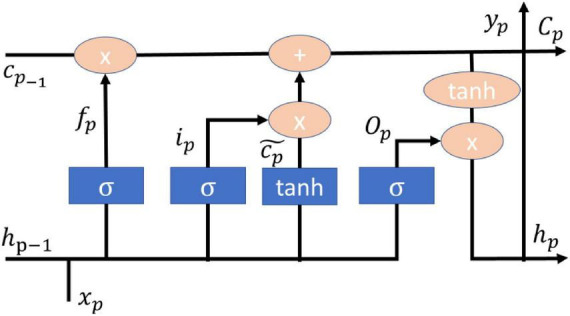
LSTM architecture.

Long short-term memory (LSTM) is a model derived from recurrent neural networks (RNN) mainly to improve the RNN memory problem, so LSTM is composed of three control valves: input valve, forgetting valve, and output valve (Hochreiter and Schmidhuber, 1997), and the LSTM model together with the dropout and l2 regularization techniques and Adam’s optimizer can achieve good diagnosis and grading in the diagnosis and severity of Parkinson’s disease (Balaji et al., 2021). The LSTM model with dropout and l2 normalization techniques and Adam’s optimizer can achieve good diagnosis and severity rating of Parkinson’s disease (Balaji et al., 2021). The number of cases and deaths in the current COVID epidemic can be predicted using LSTM model with migration learning and single or multi-step approach with good results in several countries (Gautam, 2021). Malaria is usually prevalent in subtropical countries and LSTM models can be applied to geographic location, satellite data and clinical data to achieve good prediction results (Santosh et al., 2020). Mechanical ventilation is one of the life-saving tools to help support the organs of patients with respiratory failure and improper delivery of tidal volume will lead to an increase in mortality, LSTM can achieve a level of accuracy in the prediction of tidal volume in the body (Hagan et al., 2020). The main formulas of the model of LSTM are shown in (3–8), and the architecture is shown in Figure 2.


(3) $$f_{p}=\sigma \,\left(w_{f}\cdot \left[h_{p-1},x_{p}\right]+b_{f}\right)$$

(4) $$i_{p}=\sigma \,\left(w_{i}\cdot \left[h_{p-1},x_{p}\right]+b_{i}\right)$$

(5) $$\tilde{c_{p}}=tanh\left(w_{c}\cdot \left[h_{p-1},,x_{p}\right]+b_{c}\right)$$

(6) $$C_{p}=f_{p}*c_{p-1}+i_{p}*\tilde{C_{p}}$$

(7) $$O_{p}=\sigma \,\left(w_{O}\,\left[h_{p-1},x_{p}\right]+b_{0}\right)$$

(8) $$h_{p}=O_{p}*t\,a\,n\,h\,\left(C_{p}\right)$$

*f_p_* is the forget valve, *i_p_* is the input valve, *O_p_* is the output valve, $\tilde{c_{p}}$ is the memory cell candidate. *h*_*p*–1_ is the current output value, *x_p_* is the input value. *w*_*i*_,*w*_*c*_,*w*_*o*_,*w*_*f*_ and *b*_*i*_,*b*_*c*_,*b*_*o*_,*b*_*f*_ are the weight matrix and bias vector, respectively. *C_p_* is the storage unit. σ is Sigmoid activation function.

#### 2.1.3 Gated recurrent unit

**FIGURE 3:**
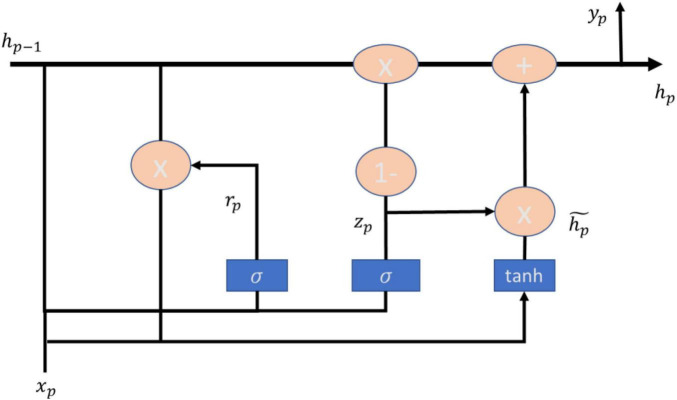
GRU architecture.

Gated recurrent unit (GRU) is a method proposed by Cho (2014), which can outperform the LSTM model in terms of CPU convergence time as well as parameter updating (Chung et al., 2014), and has been utilized by scholars in many fields such as heart failure (Gao et al., 2020), simulation of accidents at signalized intersections (Zhang et al., 2020), heartbeat detection of heartbeat signals (Hai et al., 2020). heartbeat detection with graphical signals (Hai et al., 2020) have been studied by scholars utilizing the GRU approach. GRU is an update gate used to replace the forget gate and input gate in LSTM, and then cell state and ht are merged, and the computation of GRU is also different than LSTM. The main formulas of GRU model are shown in (9–12), and the architecture is shown in Figure 3.


(9) $$z_{p}=\sigma \,\left(W_{z}*\left[h_{p-1},x_{p}\right]\right)$$

(10) $$r_{p}=\sigma \,\left(W_{r}*\left[h_{p-1},x_{p}\right]\right)$$

(11) $$\tilde{h_{p}}=t\,a\,n\,h\,\left(W*\left[r_{p}*h_{p-1},x_{p}\right]\right)$$

(12) $$h_{p}=\left(1-z_{p}\right)*h_{t-1}+z_{p}*\tilde{h_{p}}$$

### 2.2 Activation functions

#### 2.2.1 Hyperbolic tangent function

**FIGURE 4:**
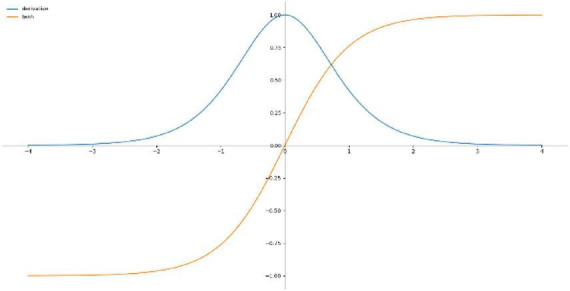
Tanh activation functions and derivatives.

Hyperbolic tangent function is a smooth and zero-centered function with the range between −1 and 1 (Nwankpa et al., 2018), and its convergence is faster and the application is similar to the Sigmoid function, so it is easier to learn slower, Figure 4 shows the Tanh activation function and derivatives, the equation is shown in (13).


(13) $$f\,\left(x\right)=\left(\frac{e^{x}-e^{-x}}{e^{x}+e^{-x}}\right)$$

#### 2.2.2 Softsign function

**FIGURE 5:**
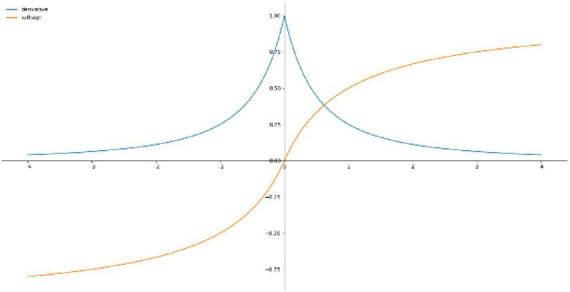
Softsign activation function and derivatives.

Softsign Function is an alternative to the hyperbolic tangent function for the reason that this model can achieve flatter curves and the derivative decreases slower and achieves a better learning method, whereas Softsign Function is decentered, differentiable and anti-symmetric and returns values between −1 and 1. Figure 5 shows the Softsign activation function and the derivative and the equation is shown in (14).


(14) $$f\,\left(x\right)=\left(\frac{x}{\left|x\right|+1}\right)$$

## 3 Materials and methods

The hardware operating system for this study is Win10 64-bit, CPU: i7, RAM: 2 4G, GPU: RTX 2070, and the software is written in Python, including Pandas, Numpy, Tensorflow, and Keras.

The research process is divided into four steps: Dataset, Data Preprocessing and Model Training and Evaluate.

### 3.1 Dataset

This study was conducted in a hospital in Taiwan from 1 August 2015 to 30 November 2020. The dataset containing the following data: extubation success or failure, Vte, RR, Ppeak, Pmean, PEEP, FiO2, and some of the collected respiratory data contained missing values. To ensure the completeness of the data, patients with missing values were excluded from this study. There were 289 sets of data for three and a half hours. In this study, there were 28 data for failed extubation and 205 data for successful extubation. There were 147 males and 86 females. The mean age was 73 years (61.8–81.3).

### 3.2 Data preprocessing

**FIGURE 6:**
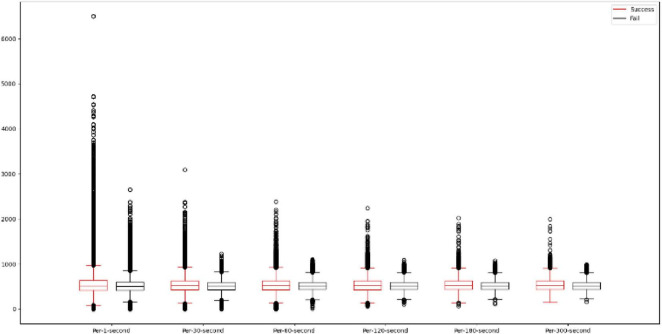
Difference in data between different averaging methods.

In this study, the data are first pre-processed by averaging the data every second, every 30 s, every 60 s, every 120 s, every 180 s, and every 300 s, and it can be seen in Figure 6 that using different averaging methods can reduce the interval between the data and also reduce the range of outliers. This study will add the original data input data with a total of 6 feature attributes, and this study will compress the input data in the range of −1 to 1 by performing the absolute maximum standardization of the input data. Since the activation functions tanh and Softsign used in this study are in the range of −1 to 1, the data compression method chosen is absolute maximum standardization instead of min-max scaling. the absolute maximum standardization formula is shown in (15).


(15) $$x_{n\,e\,w}=\frac{x}{{\left|x\right|}_{m\,a\,x}}$$

### 3.3 Model training and evaluation

Since it is difficult to collect patient data on respiratory parameters per second, this study compared three different time series deep learning models: RNN, LSTM, GRU, and four different validation methods such as: Resubstitution validation, 8:Holdout validation, 10-fold cross-validation and Leave-one-out cross-validation. Resubstitution validation, 8:2Holdout validation, 10-fold cross-validation and Leave-one-out cross-validation. Resubstitution take the first three hours of data from 250 sets of data as training samples, and the first three hours of data from 250 sets of data as validation samples, Holdout, and Cross-validation is to split the data from the first three hours of 250 sets of data into 8:2 using the Holdout method. Cross-validation is to split the data from the first 3 h of the 250 sets of data into 8:2 by using the Holdout method, the first 2 h and 24 min of the data will be used as the training sample, and the remaining 36 min will be used as the validation sample. 10-fold cross-validation is to split the data from the first 3 h of the 250 sets of data into 10-fold by using the 10-fold method, and to split the data into 10-fold by using the 10-fold method. 10-fold cross-validation is to divide the data from the first 3 h of the 250 sets of data into 10 sets of data by using the 10-fold method, one set of data is used as the validation sample and the remaining set of data is used as the training sample, and finally the 250 sets of data are divided into the first 3 h of each set of data by using the Leave-one-out method as the validation sample, and the remaining set of data is used as the first 3 h of data as the training sample. For the external validation dataset in this study, the data of each patient half an hour before extubation was used as a separate dataset. The part of the model that connects the hidden layer to the output layer uses a dropout method to prevent data over-simulation. Utilizing Accuracy in Model Evaluation for Model Evaluation. In this study, a sliding window is used to segment the time series data, and the size of the sliding window is based on every five time periods and the data are obtained in Step 1 each time, and Table 1 shows the parameter settings of the model.

**TABLE 1:** Model reference settings.

Parameter name	Parameter value
Parameter settings	Loss function: categorical_crossentropy
	Optimizer: Adam
	Learning rate: 0.006
	Epoch: 100
Model setting	Layer: 1
	Unit: 200
	Dropout: 0.2

## 4 Results

The results of this study are firstly shown in Table 2, where the Resubstitution method is utilized and then the data are averaged and pre-processed on a per-second, per-30-s, per-60-s, per-120-s, per-180-s. The results of this study are compared to the results of the model training with every 2 steps, every 3 steps, every 4 steps, and every 5 steps for training in the RNN model. Comparing the results of every 5 steps, every 3 steps, every 4 steps, and every 5 steps in the RNN model for training, the results of the study show that the training of the model with every 5 steps can get better prediction results in each average method with 76.57, 94.57, 96.59, 95.28, 91.20, and 77.67%, respectively. In per-1-s, the result of every 5 steps is slightly worse than the result of every 3 steps.

**TABLE 2:** Time step verification results.

	Time step 1	Time step 3	Time step 4	Time step 5
Per-1-s	80.09%	91.22%	90.21%	76.57%
Per-30-s	71.64%	95.10%	89.56%	94.57%
Per-60-s	71.57%	93.68%	95.57%	96.59%
Per-120-s	71.55%	89.90%	93.64%	95.28%
Per-180-s	71.80%	79.94%	88.73%	91.20%
Per-300-s	71.96%	72.53%	72.01%	77.67%

Table 3 shows the validation of the model using the activation function of tanh and different models and different validation methods for each average method, and the results show that LSTM has good prediction results in different validation methods for Per-1-s: 93.09, 91.41, 83.21, and 79.04%, and the prediction results for Per-30-s: 98.82, 94.44, 84.75, and 84.83%, for per-120-s: 10-fold. Per-30-s shows the best prediction results for GRU: 98.82, 94.44, 84.75, and 84.83%, where the 10-fold validation method is slightly inferior to LSTM, and per-60-s shows the better prediction results for LSTM: 97.75, 94.08, 84.91, and 82.30%, where the Resubstitution validation method has good prediction results, 93.09, 91.41, 83.21, and 79.04%. For per-120-s, the predictions of LSTM are 97.21, 93.16, 84.79, and 82.36%, respectively. Per-180-s has better prediction results for LSTM: 95.65, 87.35, 80.43, and 79.75%. Among them, Holdout’s validation method GRU model has better prediction results. Per-300-s comes to LSTM with better prediction results: 94.85, 88.58, 80.04, and 75.54%, respectively. Among them, Leave-one-out of the validation approach GRU model has better prediction result 79.36%, in total, the results of this study concluded that Tanh activation function performs better in the model of LSTM, while the part of average method considered that Holdout of per-30-s has better prediction result.

**TABLE 3:** Results of Tanh validation model.

	Resubstitution	Holdout cross-validation	10-fold cross-validation	Leave-one-out cross-validation
**Per-1-s**				
RNN	76.57%	72.68%	73.42%	56.73%
LSTM	93.09%	**91.41%**	**83.21%**	**79.04%**
GRU	92.62%	90.78%	82.96%	77.03%
**Per-30-s**				
RNN	94.57%	92.42%	83.33%	81.53%
LSTM	96.87%	94.22%	**85.92%**	84.68%
GRU	**98.82%**	**94.44%**	84.75%	**84.83%**
**Per-60-s**				
RNN	96.59%	89.97%	82.14%	80.92%
LSTM	97.75%	**94.08%**	**84.91%**	**82.30%**
GRU	**98.49%**	93.08%	84.12%	82.29%
**Per-120-s**				
RNN	95.28%	88.69%	80.44%	78.93%
LSTM	97.21%	**93.16%**	**84.79%**	**82.36%**
GRU	**98.18%**	93.05%	82.86%	81.66%
**Per-180-s**				
RNN	91.20%	89.47%	74.26%	77.45%
LSTM	**95.65%**	87.35%	**80.43%**	**79.75%**
GRU	95.06%	**92.04%**	79.91%	79.00%
**Per-300-s**				
RNN	77.67%	73.01%	76.89%	66.59%
LSTM	**94.85%**	**88.58%**	**80.04%**	75.54%
GRU	93.31%	84.95%	79.70%	**79.36%**

*The values in bold represent the best results at this stage.

Table 4 shows the validation of the model using the activation function as softsign and different models and different validation methods for each average method. The results show that the LSTM and GRU for per-1-s have different prediction results in different validation methods, among which Resubstitution and 10-fold have better prediction results for the LSTM model: 93.73 and 83.21%, respectively, and GRU for Holdout and Leave-one-out have better prediction results: 91.58 and 79.51% respectively. Resubstitution and 10-fold have better prediction results for LSTM model, which are 93.73 and 83.21%, respectively, and GRU has better prediction results for Holdout and Leave-one-out, which are 91.58 and 79.51%, respectively. per-30-s shows that GRU has the best prediction results for LSTM model, which are 96.71, 94.25, 86.43, and 83.46%, respectively. Resubstitution’s validation method is slightly inferior to LSTM, while per-60-s shows better prediction results for GRU: 97.97, 94.27, 84.80, and 82.16%, respectively. Per-120-s has different prediction results for different validation methods, among which Resubstitution and 10-fold validation methods have better prediction results for LSTM: 96.67 and 84.79%, respectively, and Holdout has better prediction results for RNN: 93.37%. Holdout for RNN has a better prediction result of 93.37%, Leave-one-out for GRU has a better prediction result of 81.65%, Per-180-s has different prediction results for different validation methods, among which Resubstitution and Leave-one-out for LSTM have a better prediction result of 96.82 and 80.83%, respectively. Holdout and 10-fold validation methods have better prediction results for GRU model: 92.24 and 80.96%, respectively, while Per-300-s has better prediction results for GRU: 94.59, 86.51, 81.11, and 77.85%, respectively. Among them, Holdout’s validation method LSTM model has better prediction results, and in sum, the results of this study concluded that Softsign activation function performs better in GRU’s model, and Plant’s part considered that Holdout of Plant B has better prediction results.

**TABLE 4:** Softsign validation model results.

	Resubstitution	Holdout cross-validation	10-fold cross-validation	Leave-one-out cross-validation
**Per-1-s**				
RNN	89.28%	89.32%	81.10%	74.41%
LSTM	**93.73%**	91.41%	**83.21%**	79.09%
GRU	93.22%	**91.58%**	82.53%	**79.51%**
**Per-30-s**				
RNN	95.21%	92.58%	82.77%	81.54%
LSTM	**96.75%**	94.22%	85.92%	82.73%
GRU	96.71%	**94.25%**	**86.43%**	**83.46%**
**Per-60-s**				
RNN	96.09%	90.52%	83.10%	80.79%
LSTM	97.67%	94.09%	**84.91%**	82.32%
GRU	**97.97%**	**94.27%**	84.80%	**82.61%**
**Per-120-s**				
RNN	95.69%	**93.37%**	81.15%	79.87%
LSTM	**96.67%**	93.16%	**84.79%**	80.80%
GRU	96.13%	93.32%	82.87%	**81.65%**
**Per-180-s**				
RNN	95.31%	92.14%	78.20%	78.82%
LSTM	**96.82%**	87.35%	80.43%	**80.83%**
GRU	96.20%	**92.24%**	**80.96%**	79.40%
**Per-300-s**				
RNN	93.67%	**89.79%**	81.08%	77.22%
LSTM	93.38%	88.58%	80.04%	76.71%
GRU	**94.59%**	86.51%	**81.11%**	**77.85%**

*The values in bold represent the best results at this stage.

## 5 Discussion

In this study, different averaging methods and different models were applied to compare with different activation functions, and the results of averaging every 30 s were better, and the GRU model was considered to have better predictive results in the overall assessment, and finally, Tanh’s activation function was considered to have better predictive results in terms of activation functions. In this study, Tanh’s GRU model can be effective in achieving good results regardless of success or failure and can give good advice to doctors.

In this study, different activation functions are applied for the comparison of Tanh and Softsign, in the results of this study, it can be seen that there is not much difference in the accuracy of the two activation functions and the Tanh activation function has a better predictive effect in each model.

**FIGURE 7:**
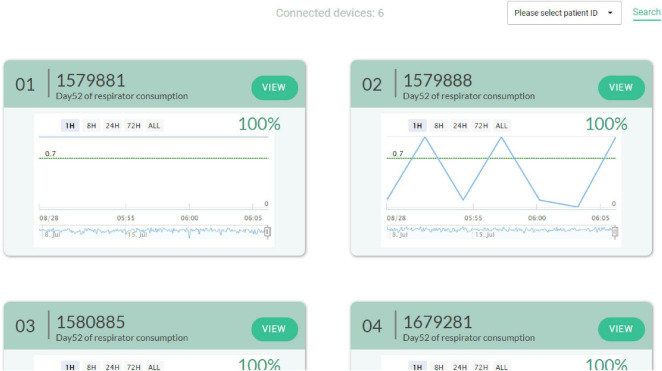
Clinical decision aids.

AI extubation decision system can be used to help the medical team to analyze whether or not to perform an extubation action before extubation. As shown in Figure 7. In this study, a trend is generated every three minutes in the clinical decision support system. If there are missing values in the data, the study will utilize the previous data to ensure that the data is complete. Therefore, the model can increase the confidence of the healthcare team in extubation. It can also reduce the burden of the patient and the family’s confidence in the medical treatment. At this stage, different countries have different laws and regulations on clinical decision-making by AI. Therefore, AIs need to strictly comply with the relevant laws in order to avoid legal disputes. The trends generated in this study can be viewed by clinicians at this time, but the right decision still needs to be made by the healthcare team.

## 6 Conclusion

The success or failure of extubation is a very important issue in medicine, and the research topic of AI is getting more and more attention. Therefore, this study compares whether the time series model can effectively predict the success or failure of extubation, and the results show that the GRU model with Tanh activation function has a better prediction result of 94.44% at the average of every 30 s, so this study proposes a prediction method using GRU on the topic of extubation, and it can provide the doctors with the clinical application of extubation to give advice for reference. In the future, this study will be possible to validate this model with several different hospitals to collect mergers from different ethnic groups to increase the credibility of the model. This model will be validated with many different hospitals to collect different groups to increase the confidence of the model. The model will also be applied to many different clinical settings for use. The data will provide by the respirator was used, and in the future, we can add more variables such as patient’s data or different expansion dimensions, and this study will use more deep learning models to verify whether they can help to achieve the prediction effect, so as to reduce the failure rate of extubation. This study will add more algorithm such as: XGBoost, LightGBM, and Transformer models for model training and comparison.

## Data Availability

The raw data supporting the conclusions of this article will be made available by the authors, without undue reservation.
